# A two disulfide bridge Kazal domain from *Phytophthora *exhibits stable inhibitory activity against serine proteases of the subtilisin family

**DOI:** 10.1186/1471-2091-6-15

**Published:** 2005-08-23

**Authors:** Miaoying Tian, Sophien Kamoun

**Affiliations:** 1 Department of Plant Pathology, The Ohio State University, Ohio Agricultural Research and Development Center, Wooster, OH 44691, USA

## Abstract

**Background:**

Kazal-like serine protease inhibitors are defined by a conserved sequence motif. A typical Kazal domain contains six cysteine residues leading to three disulfide bonds with a 1–5/2–4/3–6 pattern. Most Kazal domains described so far belong to this class. However, a novel class of Kazal domains with two disulfide bridges resulting from the absence of the third and sixth cysteines have been found in biologically important molecules, such as human LEKTI, a 15-domain inhibitor associated with the severe congenital disease Netherton syndrome. These domains are referred to as atypical Kazal domains. Previously, EPI1, a Kazal-like protease inhibitor from the oomycete plant pathogen *Phytophthora infestans*, was shown to be a tight-binding inhibitor of subtilisin A. EPI1 also inhibits and interacts with the pathogenesis-related P69B subtilase of the host plant tomato, suggesting a role in virulence. EPI1 is composed of two Kazal domains, the four-cysteine atypical domain EPI1a and the typical domain EPI1b.

**Results:**

In this study, we predicted the inhibition constants of EPI1a and EPI1b to subtilisin A using the additivity-based sequence to reactivity algorithm (Laskowski algorithm). The atypical domain EPI1a, but not the typical domain EPI1b, was predicted to have strong inhibitory activity against subtilisin A. Inhibition assays and coimmunoprecipitation experiments showed that recombinant domain EPI1a exhibited stable inhibitory activity against subilisin A and was solely responsible for inhibition and interaction with tomato P69B subtilase.

**Conclusion:**

The finding that the two disulfide bridge atypical Kazal domain EPI1a is a stable inhibitor indicates that the missing two cysteines and their corresponding disulfide bond are not essential for inhibitor reactivity and stability. This report also suggests that the Laskowski algorithm originally developed and validated with typical Kazal domains might operate accurately for atypical Kazal domains.

## Background

Proteases play essential roles in biological systems, not only digestion and protein turnover but also a diversity of specific processes [1]. To regulate the activity of proteases and avoid cellular damage, organisms also produce protease inhibitors [1]. So far, 48 distinct families of protease inhibitors have been described, one of which is the Kazal family of serine protease inhibitors (I1 family) [1]. Kazal type inhibitors are widely distributed in animals, apicomplexans and oomycetes. They are thought to play important roles in maintenance of normal cellular and physiological processes of animals [2,3], and pathogenesis of mammalian parasitic apicomplexans and plant pathogenic oomycetes [4-7]. Kazal-like serine protease inhibitors are defined by a conserved motif in their amino acid sequences. Typical Kazal domains contain six cysteine residues forming a 1–5/2–4/3–6 disulfide bond pattern [3,8]. Most Kazal domains described so far belong to this class. However, a novel class of Kazal domains has been described in recent years, in which the third and sixth cysteines are missing resulting in the loss of the 3–6 disulfide bond [3,7,9]. These two disulfide bridge domains are referred to here as atypical Kazal domains.

Atypical Kazal domains were first reported in the human serine proteinase inhibitor LEKTI, a 15-domain inhibitor associated with the severe congenital disease Netherton syndrome [2,9]. Domain 2 and 15 of LEKTI are typical Kazal domains with complete 6 cysteine residues, whereas the remaining 13 domains represent atypical two disulfide bridge Kazal domains [3,9,10]. The functionality of some atypical Kazal domains from LEKTI has been examined. Domain 1 of LEKTI does not inhibit any of the standard proteases [11]. Domain 6 exhibits significant inhibitory activity on trypsin, but this inhibition is only temporary [3,9,11]. A recombinant protein containing four atypical domains of LEKTI (domain 6, 7, 8 and 9) inhibits both trypsin and subtilisin A permanently [10], indicating that atypical Kazal domains can be effective inhibitors. However, it is unclear whether a single atypical domain can be a stable inhibitor. Multi-domain interactions could be responsible for the stable inhibitory activity observed for the recombinant protein [10]. Additional structural and functional studies on atypical Kazal domains are needed to understand the impact of the disulfide bridges on inhibitor activity and stability.

As a result of exhaustive biochemical studies of the third domain of turkey ovomucoid protein performed by the late Michael Laskowski Jr. and collaborators, much is known about the relationship between domain sequence and inhibition specificity in Kazal inhibitor-serine protease interactions. This work culminated in the development of an additivity-based sequence to reactivity algorithm, referred to from here on as the Laskowski algorithm, that predicts the inhibition constants (Ki) between Kazal domains and a set of six serine proteases based solely on the sequence of the inhibitors [12,13]. Structural studies of Kazal domain-protease complexes revealed that there are 12 contact positions (P6, P5, P4, P3, P2, P1, P1', P2', P3', P14', P15' and P18') responsible for interactions between Kazal domains and their cognate serine proteases [12,14-16]. Changes in noncontact residues often do not affect equilibrium constants (Ka, the reciprocal of Ki), whereas changes in contact residues result in significant alterations of Ka [12]. Among the 12 contact residues, P3, the second conserved cysteine residue, and P15', a conserved asparagine, show little variation in naturally occurring Kazal domains, but the remaining ten contact residues are hypervariable [12]. Therefore, the Laskowski algorithm was established based on the residues at the 10 contact positions and allows for the calculation of Ka or Ki of a Kazal domain against a selected set of six serine proteases based on the domain sequence alone [12,13,17]. This algorithm was developed based on 191 variants of turkey ovomucoid third domain (19 amino acid mutants in the ten contact residues plus the wild type) and was validated with a number of typical Kazal domains [12,13,17]. Theoretically, the algorithm should be applicable to atypical Kazal domains since the missing cysteine residues are different from the hypervariable contact residues. However, the accuracy of the algorithm in predicting the reactivity of atypical Kazal domains has not been tested (M. Laskowski, Jr., pers. comm.).

Kazal-like inhibitors are ubiquitous in oomycetes [7], a group of eukaryotic microbes that includes many devastating plant pathogens [18]. A total of 35 putative extracellular proteins with 56 predicted Kazal-like domains were identified from five plant pathogenic oomycete species [7]. Among them, the *Phytophthora infestans *Kazal inhibitors EPI1 and EPI10 inhibit and interact with the pathogenesis-related P69B subtilisin-like serine protease of the host plant tomato, suggesting a role in virulence [7,19]. Both EPI1 and EPI10 contain an atypical Kazal domain [7,19]. Atypical domains are common in Kazal-like inhibitors of plant pathogenic oomycetes. One-fourth (14/56) of oomycete Kazal domains belong to this type [7]. These domains are distributed in 14 different proteins from three *Phytophthora *species, *P. infestans*, *P. ramorum *and *P. sojae*, some of which have multiple domains. Remarkably, phylogenetic analysis of the 56 domains revealed that all the atypical domains form a significantly distinct cluster (M. Tian, Z. Liu and S. Kamoun, manuscript in preparation), suggesting that the loss of one disulfide bridge in *Phytophthora *Kazal-like domains predates speciation. Characterizing the atypical Kazal domains would help to understand the biochemical and biological functions of these inhibitors.

*P. infestans *EPI1 is an ideal candidate to characterize atypical Kazal domains. EPI1 was identified as a tight-binding inhibitor of subtilisin A and inhibits and interacts with P69B subtilisin-like serine protease [7]. EPI1 is composed of two putative Kazal domains, atypical domain EPI1a and typical domain EPI1b [7]. The predicted 12 contact residues of both domains follow the Kazal consensus, with P3 and P15' conserved cysteines and asparagines, respectively, and the remaining 10 contact residues variable relative to other Kazal domains. In this study, we predicted the inhibition constants of EPI1a and EPI1b to subtilisin A using the Laskowski algorithm [12]. The atypical domain EPI1a, but not the typical domain EPI1b, was predicted to be a strong inhibitor of subtilisin A. A recombinant EPI1a exhibited stable inhibitory activity against subilisin A and appeared solely responsible for inhibition and interaction with tomato P69B subtilase, providing evidence that the missing two cysteine residues and the corresponding disulfide bond might not be essential for inhibitor reactivity and stability. This report also suggests that the additivity-based sequence to reactivity algorithm (Laskowski algorithm) originally developed and validated with typical Kazal domains might operate accurately for atypical domains.

## Results

### The atypical Kazal domain of EPI1 is predicted to be a functional inhibitor of subtilisin A

**Figure 1:**
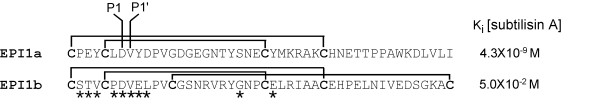
**Primary structure alignment of two Kazal domains of EPI1 and the predicted inhibition constants against subtilisin A**. The conserved cysteine residues in both domains are shown in bold. The putative P1-P1' sites and the disulfide linkages predicted based on the structure of other Kazal domains are shown. The putative 10 hypervariable contact residues are marked with asterisks. The numbers represent the inhibition constants of two Kazal domains against subtilisin A predicted with the Laskowski algorithm.

The inhibition constants of two Kazal domains of EPI1 against subtilisin A were predicted using the Laskowski algorithm [12] based on the sequence of their 10 hypervariable contact residues (Fig. 1). Interestingly, the atypical Kazal domain EPI1a was predicted to be a strong inhibitor of subtilisin A with a Ki of 4.3 nM, a value that is remarkably similar to the experimentally determined Ki of 2.77 +/- 1.07 nM for the entire EPI1 protein against subtilisin A [7]. In contrast, the typical Kazal domain EPI1b, which contains the complete set of six cysteine residues, may not be functional against subtilisin A since the predicted Ki was high at 50 mM. Therefore, these computational analyses predicted that the atypical domain EPI1a is solely responsible for the inhibition of subtilisin A.

### Expression and purification of the two Kazal domains of EPI1

**Figure 2:**
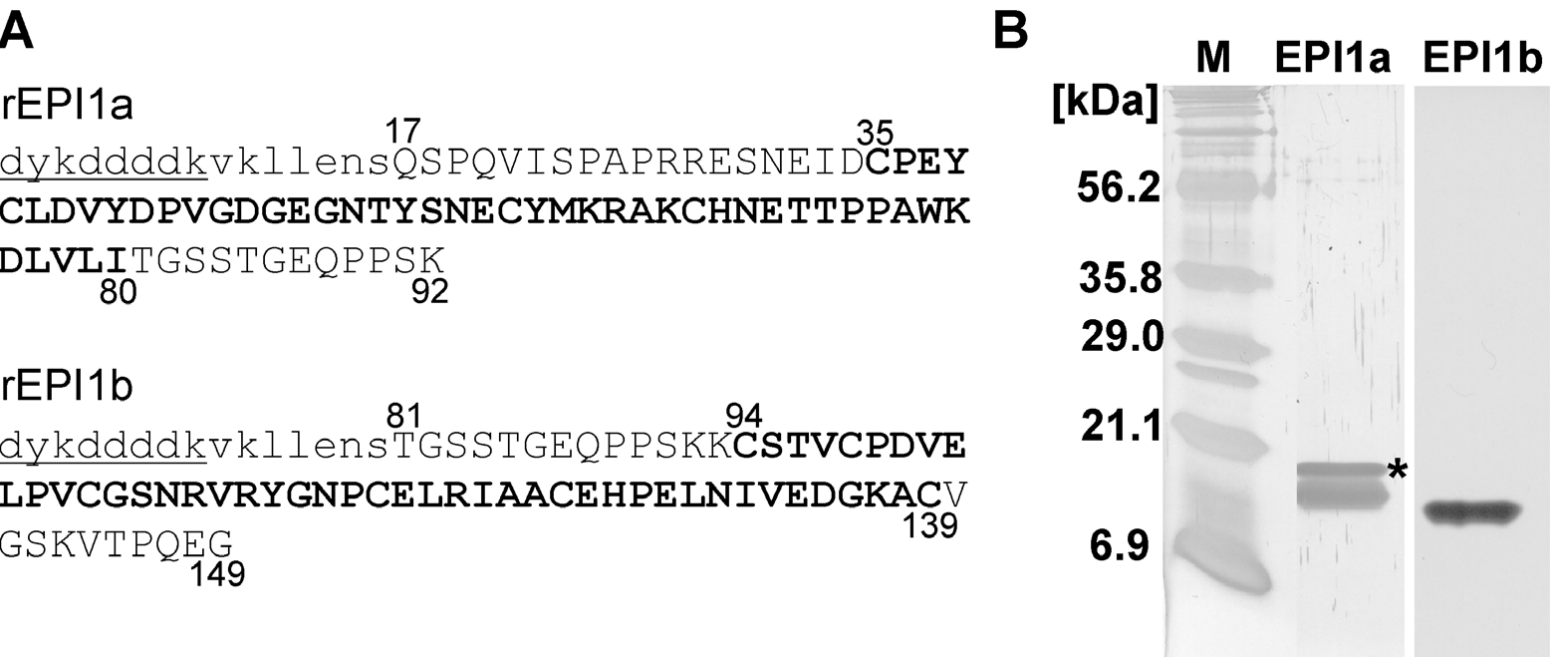
**Heterologous expression of two Kazal domains of EPI1**. A, Amino acid sequences of recombinant Kazal domains rEPI1a and rEPI1b. The letters in upper case represent the amino acid sequence of the EPI1 protein. Residues in bold correspond to the native Kazal domains EPI1a and EPI1b as shown in Fig. 1. The letters in lower case represent the vector derived sequence with the underlined ones representing FLAG epitope tag. Numbers indicate the position of amino acid residues starting from the N terminus of EPI1 protein. B, Affinity purified recombinant Kazal domains visualized on SDS-PAGE stained with silver nitrate. The rEPI1a with the signal peptide OMPA is indicated by an asterisk. The numbers on the left represent the size of molecular weight markers.

To test the protease inhibitory activities of EPI1a and EPI1b, the two Kazal domains of EPI1, and assess the predictions of the Laskowski algorithm, we expressed and purified the two recombinant domains in *Escherichia coli *as fusion proteins with the FLAG epitope tag at the amino-terminus. The sequences of the recombinant proteins are shown in Fig. 2A. The predicted molecular mass for FLAG-EPI1a (rEPI1a) and FLAG-EPI1b (rEPI1b) was 10181 Da and 8996 Da, respectively. To determine the purity of the purified recombinant proteins, we ran 0.5 μg of purified rEPI1a and rEPI1b on SDS-PAGE gel and stained with silver nitrate. Bands of the expected sizes were observed for both proteins. There was only a single band for rEPI1b, indicating high purity (Fig. 2B). The rEPI1a sample revealed two closely-migrating bands (Fig. 2B). The two bands reacted to the FLAG antibody and are likely to represent rEPI1a with and without the signal peptide OMPA, which is located immediately before the FLAG peptide in the vector pFLAG-ATS and is responsible for secreted expression in *E. coli*. Similar release of the mature protein was commonly observed with other proteins expressed using pFLAG-ATS (M. Tian and S. Kamoun, unpublished). We also stained the gel loaded with purified rEPI1a protein with Coomassie blue. Compared with the band corresponding to the rEPI1a without OMPA, the slower-migrating band was much weaker (data not shown), indicating the secreted version of rEPI1a was the major component of the purified rEPI1a protein solution. Besides these two bands, no other proteins were detected by silver staining suggesting that the rEPI1a preparation was highly pure.

### The atypical Kazal domain EPI1a inhibits the serine protease subtilisin A

**Figure 3:**
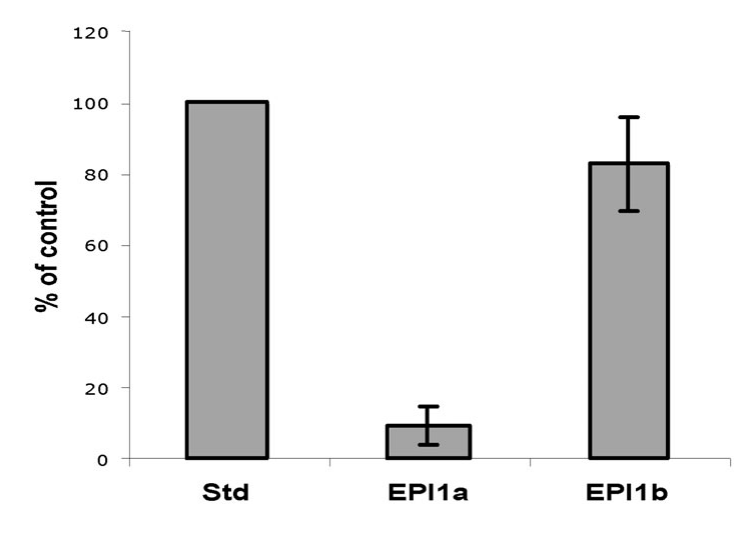
**The atypical Kazal domain EPI1a inhibits subtilisin A**. The remaining protease activity of subtilisin A was measured after incubating with rEPI1a, rEPI1b or without protease inhibitors (Std) using the QuantiCleave™ Protease Assay Kit as described in the methods. Activity is expressed as a percentage of total protease activity in the absence of protease inhibitors. The bars correspond to the mean of three independent experiments with three replications for each experiment. The error bars represent the standard errors calculated from the mean of three experiments.

We performed inhibition assays of subtilisin A by incubating 0.2 μM of subtilisin A with 0.2 μM of rEPI1a, rEPI1b or buffer control in a volume of 50 μl. The remaining protease activity was measured using the QuantiCleave™ Protease Assay Kit as described in methods. In repeated assays, rEPI1a was found to inhibit about 91% of the measured activity of subtilisin A, whereas rEPI1b did not display any significant inhibition (Fig. 3). These results are consistent with the prediction of the Laskowski algorithm (Fig. 1). It is unlikely that the FLAG tag interfered with the inhibitory activities considering that both the active rEPI1a and inactive rEPI1b carry the FLAG sequence and that these experiments were conducted in parallel.

### EPI1a inhibits the tomato pathogenesis-related P69B subtilase

**Figure 4:**
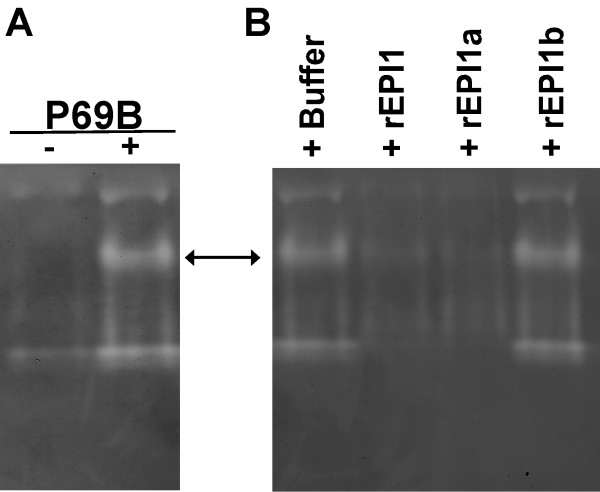
**The atypical Kazal domain rEPI1a inhibits P69B subtilase**. A, In-gel protease assay of *Nicotiana benthamiana *intercellular fluids expressing the empty binary vector pCB302-3 (-) or pCB-P69B (+). B, Inhibition assay of P69B by recombinant EPI1 entire protein and the single Kazal domains. P69B-expressing *N. benthamiana *intercellular fluids were incubated in the presence of rEPI1, rEPI1a, rEPI1b or the absence of protease inhibitors (Buffer) and then the remaining protease activity was analyzed using zymogen in-gel protease assays. The arrow indicates the band location corresponding to the protease activity of P69B.

EPI1 inhibits and interacts with the pathogenesis-related P69B subtilase of tomato [7]. To test which of the two domains of EPI1 inhibits P69B, we first used agroinfiltration to transiently express P69B fused with the epitope tag HA at the C-terminus in *Nicotiana benthamiana *leaves. Intercellular fluids were collected from leaves infiltrated with either *Agrobacterium tumefaciens *containing pCB302-P69B, or *A. tumefaciens *containing the empty binary vector pCB302-3. 10 μl of intercellular fluids from the two treatments were used in in-gel protease assays. A distinct additional protease band was observed in the P69B-expressing sample but not in the control suggesting that P69B-HA is functional (Fig. 4A). 10 μl of P69B-expressing *N. benthamiana *intercellular fluids were incubated with 20 pmol of the EPI1 recombinant protein rEPI1 [7], rEPI1a, rEPI1b, or buffer and the remaining protease activity was detected by in-gel protease assay. rEPI1a, containing the atypical Kazal domain, completely inhibited the P69B band similar to rEPI1. rEPI1 and rEPI1a also inhibited the activity of two other extracellular proteases from *N. benthamiana *(Fig. 4B). The identity of these *N. benthamiana *proteases is unknown but they could also be subtilisin-like serine proteases, such as homologs of tomato P69. In these experiments, rEPI1b did not exhibit any inhibition towards P69B or other protease bands.

### EPI1a interacts with tomato P69

**Figure 5:**
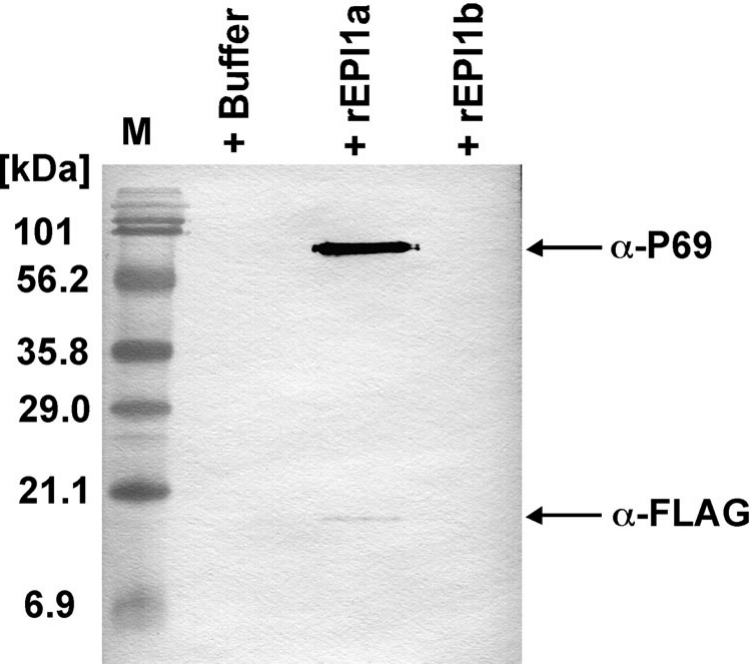
**Coimmunoprecipitation of the recombinant Kazal domains and P69 subtilases using FLAG antisera**. Eluates from coimmunoprecipitation of rEPI1a, rEPI1b or buffter with proteins in BTH-treated tomato intercellular fluids were run on SDS-PAGE gel followed by sequential immunobloting with P69 (α-P69) and FLAG (α-FLAG) antisera at a dilution of 1:3000.

We previously showed that rEPI1 interacts with P69B [7]. Here, we tested whether the atypical Kazal domain EPI1a interacts with P69B subtilase by coimmunoprecipitation. Coimmunoprecipitation was performed on BTH-induced tomato intercellular fluids incubated with rEPI1a, rEPI1b or buffer control using FLAG antibody covalently linked agarose beads. Western blots were hybridized sequentially with P69 and FLAG antisera and revealed that P69 subtilases co-precipitated with rEPI1a (Fig. 5). This indicates that rEPI1a interacts with P69 subtilases. Since the P69 family of subtilases have at least six homologs (P69A-P69F) [20,21] and the peptide used to generate the P69 antisera is conserved among several homologs [7], we cannot conclude that P69B subtilase is the only protein that was pulled down with rEPI1a. In contrast to rEPI1a, rEPI1b could not be detected after coimmunoprecipitation with BTH-induced tomato intercellular fluids (Fig. 5). However, rEPI1b was detected in control coimmunoprecipitations with the extraction buffer of tomato intercellular fluids (data not shown), suggesting that this protein is not stable in BTH-induced tomato intercellular fluids.

### EPI1a exhibits stable inhibitory activity

**Figure 6:**
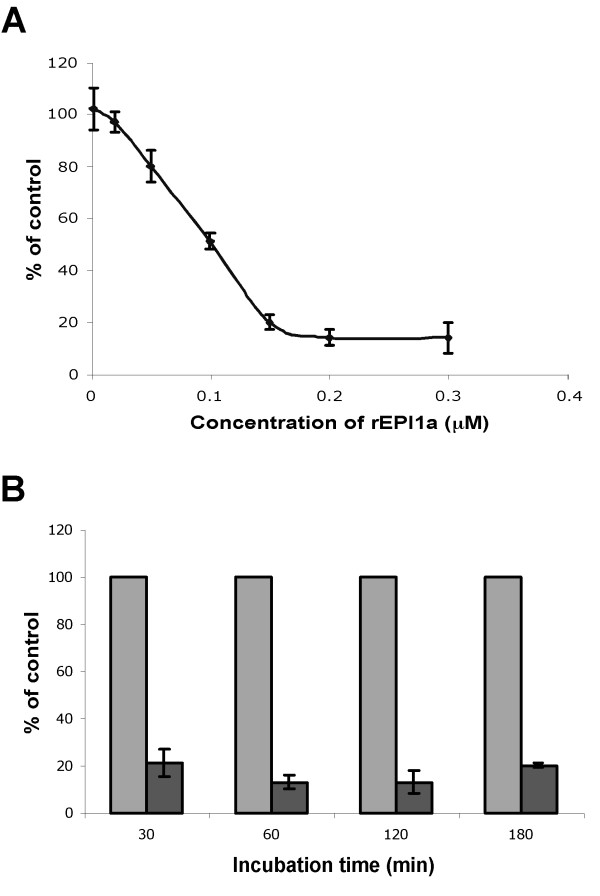
**The atypical Kazal domain rEPI1a exhibits stable inhibitory activity against subtilisin A**. A, Protease activity of subtilisin A (0.2 μM) in the presence of rEPI1a in concentrations ranging from 2 nM to 0.3 μM. Activity is expressed as a percentage of total protease activity in the absence of protease inhibitors. B, Protease activity of subtilisin A (0.2 μM) after preincubation with 0.15 μM of rEPI1a (black column) or without protease inhibitors (gray column) for a period of time ranging from 30 min to 180 min. Activity is expressed as a percentage of total protease activity in the absence of protease inhibitors at each treatment. The bars correspond to the mean of three independent replications of one representative experiment out of three performed. The error bars represent the standard errors calculated from the three replications.

To determine whether EPI1a is a temporary or stable inhibitor of subtilisin, we performed stability analyses by incubating subtilisin A with or without rEPI1a for increasing periods of time and measuring the remaining protease activity. To determine the optimal concentration of EPI1a for the stability analyses, we first performed inhibition assays with varying concentrations of EPI1a (Fig. 6A). The concentration of 0.15 μM of EPI1a resulted in inhibition levels of about 80% of the measured protease activity and was selected for the stability analysis. This concentration is within the linear part of the curve (Fig. 6A), suggesting that hydrolysis of rEPI1a could be easily detected as a decrease in inhibitory activity. The inhibitory activity of rEPI1a did not show any decrease over 3 hours of incubation with subtilisin A (Fig. 6B), indicating that EPI1a is a stable inhibitor of subtilisin A. These results are in sharp contrast with those reported for domain LD-6 of LEKTI, which lost 50% of inhibitory activity after 1 hour of incubation with trypsin and lost all inhibitory effect after 3–4 hours [3].

## Discussion

Atypical Kazal domains with two disulfide bridges occur in biologically important molecules. The 15-domain human serine protease inhibitor LEKTI that carries 13 atypical Kazal domains is associated with the severe congenital disorder Netherton syndrome [2]. The protease inhibitors EPI1 and EPI10 of *P. infestans *contain an atypical Kazal domain and have been implicated in virulence of this devastating plant pathogen [7,19]. Although the structure and function of atypical Kazal domains from LEKTI have been studied, the effects of the loss of Cys 3, Cys 6 and the corresponding disulfide bond on inhibitor reactivity and stability was not assessed and there is no evidence showing that a single atypical Kazal domain can be a stable inhibitor by itself [3,10,11]. In this study, we describe that the atypical domain EPI1a of the two-domain EPI1 protein is a stable inhibitor of the subtilisin family of serine proteases. No loss of inhibitory activity was found even after incubating EPI1a with subtilisin A for 3 hours. The loss of Cys 3, Cys 6 and the corresponding disulfide bond does not have major adverse effects on inhibitory activity or stability, indicating that these two cysteine residues might not be essential for the function of Kazal domains. This finding is important for determining the biochemical and biological functions of Kazal inhibitors containing atypical Kazal domain(s). Kazal-like proteins have been reported from animals, apicomplexans, oomycetes, as well as the bacterium *Nitrosomonas europaea *[7]. The availability of genomic sequence from a diversity of organisms is likely to reveal an increasing number of atypical Kazal inhibitors. For example, so far, a total of 14 atypical Kazal domains have been identified in plant pathogenic oomycetes [7].

The structural mechanism underlying the stability of EPI1a is not clear. The three-dimensional structure of the atypical domain 6 (LD6) of LEKTI was determined. The overall structure of LD6 resembles the three-dimensional fold of typical Kazal-type inhibitors, but the backbone geometry of its canonical loop is not well defined, providing a possible explanation for its temporary inhibitory activity [11]. There are 13 residues between the first cysteine residue (Cys 1) and the second one (Cys 2) in LD6, instead of 6–9 in most typical Kazal domains [11]. The lack of one disulfide bond and the longer sequence stretch between the first two cysteines were proposed to be the factors responsible for the instability of LD6 [11]. Indeed, the longer sequence stretch between the first two cysteines could explain the difference between EPI1a and LD6. There are only 3 residues between Cys 1 and Cys 2 in EPI1a, which is shorter than in most Kazal domains. The longer sequence stretch of LD6 might lead to the abnormal canonical loop and the non-permanent inhibitory activity. Future work to determine the three-dimensional structure of EPI1a and compare it with LD6 of LEKTI and other Kazal domains should help to unravel the structural mechanism underlying the functionality of atypical Kazal domains.

The Laskowski algorithm was developed and validated based on typical Kazal domains [12,13]. The algorithm exploits an exhaustive analysis of all amino acid variants in the ten hypervariable contact residues of turkey ovomucoid third domain, a typical Kazal domain. The accuracy of the algorithm in predicting the reactivity of atypical Kazal domains has not been evaluated (M. Laskowski Jr., pers. comm.). Here we found that the algorithm correctly predicted which of the two EPI domains is likely to inhibit subtilisins. Our experimental data showed that the atypical Kazal domain EPI1a inhibited subtilisin A, and inhibited and interacted with P69 subtilase similar to the entire EPI1 protein [7]. Also, using the algorithm, the atypical Kazal domain EPI1a was predicted to be a strong inhibitor of subtilisin A with a predicted Ki of 4.3 nM, which was in very good agreement with the experimentally determined Ki of 2.77 +/- 1.07nM [7]. As expected from the predicted Ki of 50 mM, the typical EPI1b domain was not an effective inhibitor of subtilisin A. In summary, it appears that the Laskowski algorithm operates accurately for atypical Kazal domains such as EPI1a. Perhaps, this is expected since the Cys 3 and Cys 6 residues of typical Kazal domains are not contact positions. Nonetheless, our observations and the concordance between predicted and experimental data suggest that gross structural changes that could result from the loss of one disulfide bridge in atypical Kazal domains may not affect the specificity of the interactions between Kazal domains and their cognate serine proteases.

Atypical Kazal domains are ubiquitous in serine protease inhibitors of plant pathogenic oomycetes. Fourteen of a total of 56 Kazal-like domains identified in five plant pathogenic oomycetes have only four cysteines. Two of these Kazal-like inhibitors EPI1 and EPI10 of *P. infestans *target the defense-related protease P69B of the host plant tomato. The first atypical Kazal domain of EPI1 appears to be solely functional in inhibiting and interacting with P69B. In the three-domain EPI10, the second domain is also an atypical domain that was predicted to be functional against subtilisin based on the Laskowski algorithm [19]. These findings raise some interesting questions. What are the biochemical and biological implications of the loss of the disulfide bridge? Are there any evolutionary advantages of the two-disulfide bridge Kazal domain over the three-disulfide bridge domain in counteracting and co-evolving with host proteases? Additional functional and structural studies are needed to address these questions.

## Conclusion

In this study, the functionality of a two disulfide bridge atypical Kazal domain EPI1a from *Phytophthora *was characterized. EPI1a was predicted to be a strong inhibitor of subtilisin A using the additivity-based sequence to reactivity algorithm (Laskowski algorithm). Inhibition assays and coimmunoprecipitation experiments showed that recombinant domain EPI1a exhibited stable inhibitory activity against subilisin A and was solely responsible for inhibition and interaction with tomato P69B subtilase, providing evidence that the missing two cysteines and their corresponding disulfide bond are not essential for inhibitor reactivity and stability. This report also suggests that the Laskowski algorithm originally developed and validated with typical Kazal domains might operate accurately for atypical Kazal domains.

## Methods

### Prediction of inhibition constants

The putative ten hypervariable contact residues of EPI1a and EPI1b were identified based on similarity to canonical animal Kazal domains [14-16] and are shown in Fig. 1. Predicted inhibition constants for the EPI1 domains against subtilisin A (Carlsberg) were generated by Drs. M. A. Qasim and M. Laskowski Jr., Purdue University, with the additivity-based sequence to reactivity algorithm (Laskowski algorithm) described by Lu et al. [12].

### Plant growth and BTH treatment

Tomato (*Lycopersicon esculentum*) cultivar Ohio 7814 and *N. benthamiana *plants were grown in pots at 25°C, 60% humidity, under 16 hour-light/8 hour-dark cycle. We used the salicylic acid analog benzo-(1,2,3)-thiadiazole-7-carbothioic acid S-methyl ester (BTH) to induce PR proteins. BTH treatment of tomato plants followed the exact same procedure described previously [7].

### Bacterial strains and plasmids

*E. coli *XL1-Blue and *A. tumefaciens *GV3101 were used in this study and were routinely grown in Luria-Bertani (LB) media [22] at 37°C and 28°C, respectively. Plasmids pFLAG-EPI1a and pFLAG-EPI1b for protein expression were constructed by cloning the PCR amplified DNA fragments corresponding to the coding sequence of Kazal domains EPI1a and EPI1b together with some flanking sequence into *Eco*RI and *Kpn*I sites of pFLAG-ATS (Sigma, St. Louis, MO), a vector that allows secreted expression in *E. coli*. The primers used for amplification of *epi1a *are epi1a-F1(5'-gcggaattcTCAAAGCCCGCAAGTCATCAG-3') and epi1a-R1(5'-gcgggtaccTTACTTGCTGGGAGGCTGCTCGCCAG-3'). The primers used for amplification of *epi1b *are epi1b-F1(5'-gcggaattcCACCGGTAGCTCCACTGGCGAGCAGC-3') and epi1b-R1(5'-gcgggtaccTTATCCCTCCTGCGGTGTC-3'). The introduced *Eco*RI and *Kpn*I restriction sites for cloning are underlined. The letters in upper case represent gene specific sequence. The detailed sequence information for the expressed fusion proteins FLAG-EPI1a (rEPI1a) and FLAG-EPI1b (rEPI1b) is shown in Fig. 2A. Plasmid pCB-P69B is a construct with the open reading frame of the *P69B *gene [GenBank: Y17276] fused with the HA tag (YPYDVPDYA) at the C-terminus cloned into the binary vector pCB302-3 [23] and was described elsewhere [19].

### SDS-PAGE and Western blot analyses

Proteins were subjected to 15% sodium dodecyl sulfate-polyacrylamide gel electrophoresis (SDS-PAGE) as previously described [22]. Following electrophoresis, gels were stained with silver nitrate following the method of Merril et al. [24] or with Coomassie Brilliant Blue [22], or the proteins were transferred to supported nitrocellulose membranes (BioRad Laboratories, Hercules, CA) using a Mini Trans-Blot apparatus (BioRad Laboratories, Hercules, CA). Detection of antigen-antibody complexes was carried out with a Western blot alkaline phosphatase kit (BioRad Laboratories, Hercules, CA). Antisera to P69 subtilases were raised against a peptide specific for the tomato P69 family [7]. Monoclonal anti-FLAG M2 antibody was purchased from Sigma (St. Louis, MO).

### Expression and purification of rEPI1a and rEPI1b

Expression and purification of rEPI1a and rEPI1b was conducted as described previously for other pFLAG-ATS derived constructs [7,25]. Protein concentrations were determined using the BioRad protein assay (BioRad Laboratories, Hercules, CA). To determine the purity, 0.5 μg of the purified protein was run on a SDS-PAGE gel followed by staining with silver nitrate.

### Transient expression of P69B subtilase in planta

Transient expression of P69B-HA *in planta *was performed according to the agroinfiltration method described previously [26]. *A. tumefaciens *strains carrying plasmids pCB-P69B, empty vector pCB302-3 [23], and pCB301-P19 (J. Win and S. Kamoun, unpublished) were used. pCB301-P19 is a construct expressing the P19 protein of tomato bushy stunt virus (TBSV), a suppressor of post-transcriptional gene silencing in *N. benthamiana *that significantly enhances *in planta *transient expression [27]. Overnight agrobacteria cultures were harvested by centrifugation at 2000 g for 20 min, and resuspended in 10 mM MgCl_2_, 10 mM MES (pH 5.6) and 150 μM acetosyringone. Resuspended agrobacteria cultures of pCB-P69B or pCB302-3 with an optical density (OD_600_) of 1.0 were mixed with equal volumes of a culture of pCB301-P19 with an optical density (OD_600_) of 2.0. The mixtures were kept at room temperature for 3 hours and then infiltrated into leaves of 6-week-old *N. benthamiana *plants. Intercellular fluids from infiltrated leaves were isolated 5 days after infiltration.

### Isolation of intercellular fluids

Intercellular fluids were prepared from tomato and *N. bethamiana *leaves according to the method of de Wit and Spikman [28]. For tomato leaves, a 0.24 M sorbitol solution was used as extraction buffer. For leaves from *N. benthamiana*, a solution of 300 mM NaCl, 50 mM NaPO_4 _pH 7 [26] was used as extraction buffer. The intercellular fluids were filter sterilized (0.45 μM), and were used immediately or stored at -20°C.

### In-gel protease assays

In-gel protease assays were performed with 10% SDS-polyacrylamide gel containing 0.1% (w/v) gelatin (BioRad Laboratories, Hercules, CA) using BIO-RAD's zymogram buffer system as described earlier [7].

### Inhibition assays of subtilisin A by EPI1 Kazal domains

Inhibition assays of subtilisin A by EPI1 Kazal domains were performed using colorimetric QuantiCleave™ Protease Assay Kit (Pierce, Rockford, IL). 0.2 μM of subtilisin A (Carlsberg) (Sigma, St. Luis, MO) was preincubated with different amount of purified EPI1 Kazal domains, in a volume of 50 μl buffer for 30 min at 25°C, and then the remaining protease activity was measured following the procedures as described previously [7]. Analysis of the stable inhibitory activity of rEPI1a against subtilisin A was performed by incubating 0.2 μM of subtilisin A with 0.15 μM of rEPI1a in 50 μl buffer (50 mM Tris, pH 8.0) for a time period of 0–180 min at 25°C and then measuring residue enzyme activity.

### Coimmunoprecipitation

Coimmunoprecipitation of Kazal domains rEPI1a and rEPI1b with BTH-treated tomato intercellular fluids was performed using the FLAG-tagged protein immunoprecipitation kit (Sigma, St. Luis, MO) as described previously [7]. 100 pmol of purified rEPI1a or rEPI1b were preincubated with 300 μl of tomato intercellular fluids for 30 min at 25°C. 40 μl of anti-FLAG M2 resin was added and incubated at 4°C for 2 h with gentle shaking. The precipitated protein complexes were eluted in 60 μl of FLAG peptide solution (150 ng/μl) and were analyzed by SDS-PAGE and Western blot analyses.

## Authors' contributions

MT, designing and performance of wet lab experiments, writing of manuscript. SK, supervision of experimental work, writing of manuscript.
